# An Unusual Presentation of Postpartum Spontaneous Coronary Artery Dissection

**DOI:** 10.5811/cpcem.2019.4.41305

**Published:** 2019-05-20

**Authors:** Jonathan Alterie, Francis Villanueva, Mohamed Arekat, April Brill

**Affiliations:** * Chicago College of Osteopathic Medicine, Department of Emergency Medicine, Downers Grove, Illinois; † Chicago College of Osteopathic Medicine, Downers Grove, Illinois; ‡ Franciscan Health, Department of Cardiology, Olympia Fields, Illinois

## Abstract

The postpartum population is one with a unique physiologic profile that predisposes these patients to rare and often life-threatening conditions. Herein, we discuss a case of a 37-year-old, multiparous female who presented to the emergency department with vague chest discomfort 14 days after delivering her sixth child via vaginal delivery. The patient was found to have elevated cardiac biomarkers and was ultimately diagnosed with pregnancy-related spontaneous coronary artery dissection (P-SCAD). This case report discusses the evaluation, pathophysiology, workup, and management of P-SCAD.

## INTRODUCTION

Pregnancy-related spontaneous coronary artery dissection (P-SCAD) is a rare complication in the postpartum patient, but it is the most common cause of acute coronary syndrome (ACS) in young females without coronary artery disease.^1^,^2^ Those suffering from P-SCAD typically present with signs and symptoms similar to ACS.^2^,^3^ We report a case of a postpartum female who presented to our emergency department (ED) with atypical signs of ACS and ultimately was diagnosed with P-SCAD. This case exemplifies the need for the emergency physician to keep a broad differential diagnosis for the postpartum patient as these patients comprise a unique population and may not always present in a typical manner.

## CASE REPORT

A gravida 6, para 6, 37-year-old, well-appearing female presented to our ED approximately two weeks postpartum after a term, singleton vaginal delivery with a chief complaint of non-specific chest pain for one week. Her chest pain was substernal, intermittent, sharp, and non-radiating. It was exacerbated with certain movements and positions such as picking up her children and lying supine. Associated symptoms were shortness of breath and palpitations. Her pregnancy was complicated by gestational diabetes requiring subcutaneous insulin therapy and iron deficiency anemia requiring transfusion of one unit of packed red blood cells.

The patient’s family history was significant for a brother who required a cardiac stent for coronary artery disease at the age of 40 and her mother who developed cardiovascular disease later in life. The patient’s physical exam and vital signs were unremarkable. However, given her family history of early onset of coronary artery disease and being 14 days postpartum, we pursued a cardiopulmonary workup. The initial workup was notable for an electrocardiogram (ECG) that demonstrated normal sinus rhythm with left axis deviation and a chest radiograph that revealed trace pleural effusions with an enlarged cardiac silhouette. Labs were significant for microcytic anemia: hemoglobin 10.5 grams per deciliter (g/dL) (12.1–15.1 g/dL) and hematocrit 33% (34.9–44.5%) and an initial troponin I elevated at 0.92 nanograms per milliliter (ng/mL) (<0.03 ng/mL). A repeat troponin I was 2.81ng/mL. A chest computed tomography angiography (CTA) did not reveal pulmonary embolism but confirmed pleural effusions. Cardiology was consulted and elected to take the patient for angiogram due to the rising troponin and negative CTA.

**Image 1:**
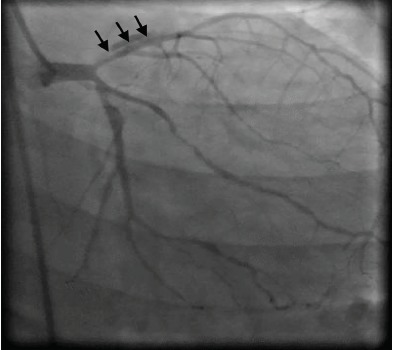
Selective left coronary angiogram in the right anterior coronary oblique caudal projection showing the proximal portion of a spontaneous dissection in the left anterior descending artery (arrows).

**Image 2:**
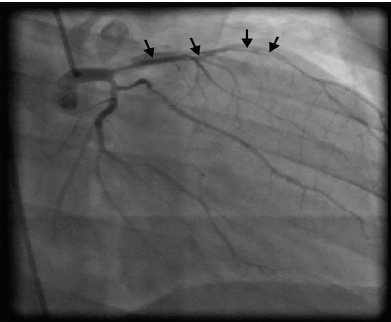
Right anterior oblique caudal projection showing the proximal portion of a spontaneous dissection in the left anterior descending artery extending to the junction of the middle and distal thirds of the left anterior descending artery (arrows).

Coronary angiogram revealed a spontaneous dissection extending to the junction of the distal third left anterior descending artery (Images 1 and 2). Due to the extensive nature of the dissection, the interventional cardiology team consulted cardiothoracic surgery, which deemed it more appropriate that the patient undergo coronary artery bypass grafting (CABG). It was intraoperatively that she was found to have bilateral spontaneous coronary dissections from the ostia of both the left main and the right main coronary arteries all the way to the distal end of all of the coronary tree. A five-vessel CABG was performed successfully and the patient was discharged postoperative day six.

**CPC-EM Capsule**

What do we already know about this clinical entity?

Increased cardiovascular risk and anomalies are linked to the pregnant and postpartum states due to the hormonal and hemodynamic demands of pregnancy.

What makes this presentation of disease reportable?

This presentation is reportable as the patient presented with rather vague symptoms, worse with movement, as opposed to an acute onset with “tearing” chest pain.

What is the major learning point?

Pregnant and post-partm spontaneous coronary dissection may present variably and with vague symptoms. It is important to understand this unique population.

How might this improve emergency medicine practice?

This may result in faster identification of the disease process and lead to quicker intervention and better outcomes for pregnant or postpartum patients.

## DISCUSSION

The term dissection refers to the separation of the arterial wall layers, creating an additional channel for blood to flow, which is called a false lumen. This false lumen can collapse upon the true lumen causing occlusion.^2^–^5^ Coronary artery dissection can be characterized as primary (spontaneous) or secondary, the latter being more common.^4^ It has been cited that SCAD has a strong association with pregnancy and those suffering from connective tissue disorders.^2^,^4^,^5^,^6^ Although the exact cause of P-SCAD is still under investigation, studies postulate that the physiological cardiac demands of pregnancy, hormonal changes in estrogen and progesterone, and the hemodynamic strains of labor and delivery contribute to the development of intimal wall tears and degeneration within coronary artery walls. Despite having some cardioprotective effects, estrogen may play a role in upregulating the release of matrix metalloproteinases and contributing to the loss of structural support. Increased progesterone levels weaken the arterial wall by disrupting the normal corrugation of elastic fibers and degradation of medial wall collagen. Recurrent exposures to high levels of estrogen and progesterone, as seen in multiparity, can have additive effects to the degeneration of the arterial wall.^2^,^3^,^4^,^7^

Although P-SCAD can affect women considered peripartum, the highest incidence occurs during the postpartum period. According to a retrospective review of the Mayo Clinic’s SCAD registry by Tweet et al., nearly all of the women diagnosed with P-SCAD presented within the 12 weeks following delivery, and the greater majority within one week postpartum.^1^,^7^ When compared to other women of childbearing age in the United States. population, those who developed P-SCAD were more likely to have suffered from pre-eclampsia and had undergone treatment for infertility. They were also more likely to be multiparous; however, there was no significant difference between these two groups in terms of number of childbirths. Only a small percentage of those suffering from P-SCAD showed risk factors or predisposing health conditions that are typical of non-pregnancy related SCAD, such as fibromuscular dysplasia, antiphospholipid syndrome, Marfan syndrome, connective tissue dysplasia, and Ehlers-Danlos syndrome.^1^,^3^

The most common signs and symptom are those similar to ACS.^1^–^3^ According to one study, which compiled data from the three largest literature reviews of P-SCAD, the most common presentations included chest pain, dyspnea, acute myocardial infarction, congestive heart failure, ventricular arrhythmia, sudden cardiac death, and cardiogenic shock.^3^ Eliciting a medical history will often show no other predisposing risk factors for cardiovascular disease other than the patient being postpartum.^8^

Findings consistent with P-SCAD are ST-segment elevation myocardial infarction (STEMI), non-STEMI, left main or multivessel SCAD, or a left ventricular dysfunction of less than 35%.^3^ Workup may demonstrate ischemic changes on ECG, pericardial tamponade, or elevated cardiac biomarkers.^3^,^6^ In a majority of cases, ECG will show ST-segment changes in a left coronary artery system distribution.^1^,^8^ Diagnosis is confirmed with coronary angiography as CTA has inadequate spatial resolution to visualized SCAD in smaller coronary arteries.

Current management of P-SCAD is controversial and without clear guidelines.^1^,^2^ P-SCAD may be treated with conservative therapy, percutaneous coronary intervention (PCI), temporizing measures such as extracorporeal membrane oxygenation or CABG surgery for the most severe cases.^7^–^12^ Conservative management may be considered in patients who have no evidence of ongoing ischemia and no significant stenosis on cardiac catheterization. This management includes the use of heparin, beta blockers, calcium channel blockers, nitrates, and antiplatelet therapy. However, these therapies are often only short-term solutions. PCI is often the treatment of choice for involvement of a single vessel and CABG is preferred in a patient with multi-vessel dissection, complex lesions, or failed PCI.^3^ Treatment is chosen on a case by case basis depending on severity of the dissection(s), amount of salvageable myocardium, and the overall clinical presentation.

This case report brings to light a postpartum female with multiple risk factors for P-SCAD who presented with vague and poorly explained chest pains. We pursued a workup due to her risk factors, which placed her into a higher risk subpopulation. The purpose of this case report is to highlight the need for recognition of risk factors for a certain condition such as P-SCAD as these factors may sometimes (as in our case) be the only heralding findings of a case that spurs a workup in a certain direction.

## CONCLUSION

P-SCAD is a rare condition that is under-represented in the emergency medicine literature. The patient discussed in our case represents a novel presentation of P-SCAD. Without obtaining a thorough and complete history, a clinician may have easily misdiagnosed her with reflux or postpartum musculoskeletal pain. This case demonstrates that not all patients with P-SCAD present with obvious signs and symptoms of ACS. The threshold to work up a postpartum female presenting with poorly explained symptoms of acute coronary syndrome should be low, and P-SCAD should be on the differential no matter how innocuous the presentation.
